# Commentary: Lomustine-temozolomide combination therapy versus standard temozolomide therapy in patients with newly diagnosed glioblastoma with methylated *MGMT* promoter (CeTeG/NOA-09): a randomised, open-label, phase 3 trial

**DOI:** 10.3389/fonc.2020.00066

**Published:** 2020-01-31

**Authors:** Sunit Das, Arjun Sahgal, James R. Perry

**Affiliations:** ^1^Division of Neurosurgery and Li Ka Shing Knowledge Institute, St. Michael's Hospital, University of Toronto, Toronto, ON, Canada; ^2^Department of Radiation Oncology, Sunnybrook Hospital, University of Toronto, Toronto, ON, Canada; ^3^Division of Neurology, Sunnybrook Hospital, University of Toronto, Toronto, ON, Canada

**Keywords:** glioblastoma, chemotherapy, temozolomide, lomustine (CCNU), radiation therapy

The introduction of concurrent temozolomide (TMZ) to radiation therapy (RT) by the EORTC-NCIC trial (the Stupp trial) was a significant advance in the treatment of adult patients with glioblastoma (1). In Stupp, adult patients with newly diagnosed glioblastoma who received radiation with concurrent and adjuvant TMZ had a median overall survival (OS) of 14.6 months, compared to 12.5 months with RT alone. More significantly, patients with glioblastoma for the first time began to achieve longer-term survival, with nearly 40% alive at 2 years, and nearly 10% alive at 5 years (2).

Soon thereafter, in a retrospective analysis of data from the Stupp trial, Hegi et al. showed that benefit from TMZ chemotherapy was largely limited to patients with glioblastoma possessing a methylated O-6-methylguanine-DNA methyltransferase (MGMT) promoter (3). In fact, these data suggested that MGMT promoter methylation might be a clinically relevant predictor of benefit from TMZ chemotherapy, and conversely, that unmethylated MGMT promoter status might be a clinically relevant predictor for lack of efficacy for TMZ.

MGMT expression was first proposed to be a resistance factor in glioma in the 1990s, following mechanistic findings from the laboratory identifying a role for MGMT in DNA repair following alkylating agent-mediated injury. Subsequently, Esteller et al., in an analysis of samples harvested from glioma patients treated with the alkylating agent carmustine (BCNU), demonstrated a correlation between MGMT promoter methylation and tumor response and overall survival (4). Soon thereafter, this correlation was also found for TMZ (5). Since then, numerous trials have demonstrated the prognostic effect of MGMT status on survival in patients with newly diagnosed glioblastoma. As these trials have all involved upfront treatment with TMZ, it has not been possible to use these data to determine if MGMT status is predictive of survival. With two exceptions: the Nordic trial and NOA-08, two randomized trials comparing single-agent TMZ vs. RT in elderly patients, both of which confirmed MGMT status to predict benefit from TMZ (6, 7). What is clear from the data available to us is that patients harboring a tumor with a methylated MGMT promoter survive longer and likely respond more robustly to TMZ chemotherapy.

New data from Herrlinger et al. reinforce a conclusion that chemotherapy is effective in patients with methylated promotor status (8). This study was driven by findings from UKT-03 trial, a small single–arm phase 2 trial (31 patients) that explored combined lomustine/TMZ chemotherapy in patients with newly diagnosed glioblastoma (9, 10). In contrast to TMZ, lomustine has effects beyond DNA alkylation: it also introduces inter-strand crosslinks and leads to carbamoylation of amino acids, both of which interfere with transcriptional, translational, and post-transcriptional processes (11). As in previous trials using nitrosoureas, the first course of chemotherapy started during RT. Median OS was 23 months with lomustine/TMZ combination therapy, compared to 15–17 months in contemporary historical controls. Notably, the OS benefit was only seen in patients with methylated MGMT promoter: median OS was 12.5 months in patients with an unmethylated MGMT promoter. Median OS of patients with methylated MGMT was 34.5 months, comparing favorably to 23.4 months in patients with methylated MGMT status who received TMZ chemoradiation in the Stupp trial (3).

CeTeG/NOA-09 was an open-label phase III trial conducted at seventeen German university hospitals. Patients aged 18–70 years with newly diagnosed methylated MGMT promoter glioblastoma with a Karnofsky performance status score >70 were considered for study. Patients were randomly assigned (1:1) to two arms: (1) TMZ chemoradiotherapy [75 mg/m^2^ per day concomitant to radiotherapy (59–60 Gy)] followed by six courses of TMZ (150–200 mg/m^2^ per day on the first 5 days of the 4-week course); or (2) up to six courses of lomustine (100 mg/m^2^ starting on day 1) plus TMZ (100–200 mg/m^2^ per day on days 2–6, of the 6-week course) in addition to RT (59–60 Gy) with 5 days of concomitant TMZ chemotherapy (150 mg/m^2^ per day). Because of the different schedules, patients and physicians were not masked to treatment groups. The primary endpoint was defined as OS in the modified intention-to-treat population (all randomly assigned patients who started their allocated chemotherapy).

From June 17, 2011, to April 8, 2014, CeTeG/NOA-09 enrolled 141 patients. One hundred twenty-nine patients (63 in the TMZ and 66 in the lomustine-TMZ group) constituted the modified intention-to-treat population. Median OS was 31.4 months (95% CI 27.7–47.1) in the TMZ group, compared to 48.1 months (32.6 months–not assessable) in the lomustine-TMZ group, for a hazard ratio [HR] of 0.60 (95% CI 0.35–1.03; *p* = 0·0492). Progression-free survival (PFS) did not differ between the treatment groups in the modified intention-to-treat population (*p* = 0.4113, stratified log-rank test) or in the intention-to-treat population (*p* = 0.4735). Adverse events of grade 3 or higher were observed in 32 of 63 patients (51%) in the TMZ group compared to 39 of 66 patients (59%) in the lomustine-TMZ group. There were no treatment-related deaths. Based on these findings, the authors conclude that that combined lomustine-TMZ therapy might be better than standard TMZ therapy in patients with newly diagnosed methylated MGMT promoter GBM.

The CeTeG/NOA-09 trial has significant limitations. Of the 653 patients screened only 234 (36%) were found to have MGMT promoter methylation; of these only 141 patients were randomized into the trial. Reasons for non-inclusion were patients wish, not meeting inclusion criteria, administrative reasons, or unknown reasons in descending order of frequency. As the authors admit, this small number of patients ultimately participating limits the generalizability (external validity) of the results and leaves study open to bias from known, and unknown, prognostic variables.

Another limitation was the discrepancy between the improvement in OS and the absence of an effect on PFS. This discrepancy was not based on differences in treatment at recurrence/progression: re-irradiation and further treatment with other chemotherapies were no more frequent in the TMZ standard treatment group than in the lomustine-TMZ group. Further, rates of repeat resection and use of anti-angiogenic therapies were similar in the two groups. The authors hypothesize that an increased prevalence of late and prolonged radiographic pseudoprogression after lomustine-TMZ therapy might have had a major role in the discrepancy between OS and PFS; in other words, that PFS for the lomustine-TMZ group was underestimated because of the inclusion of a greater proportion of patients with pseudoprogression as progressors, a conclusion supported by the observation that most pseudoprogressions with lomustine-TMZ (six of seven with lomustine-TMZ vs. two of five with TMZ) were defined only by histology.

Another explanation could be that undetected pseudoprogression was particularly prevalent in the first 2 years after the start of therapy, thus providing an explanation for the late separation of the PFS curves after 2 years. It is difficult to speculate on the issue of pseudoprogression in this trial because of the difference in RT between the arms; the TMZ arm received standard chemoradiation with concurrent TMZ and adjuvant TMZ, while the combination arm received only a short course (5 days) of concurrent TMZ with RT. Pseudoprogression is likely more common following RT/TMZ than RT alone (although this assumption is controversial) but this limits interpretation of results and comparison to other contemporary trials where RT with concurrent TMZ is standard. Notably the deliberate shortening of the concurrent TMZ course in the study arm actually creates two interventions in this study: short-course concurrent TMZ and the addition of CCNU to standard adjuvant therapy. Practitioners considering adopting this adjuvant regimen in practice should be aware this study does not address the toxicity of TMZ-CCNU combination therapy in patients who receive standard TMZ chemoradiation. It is interesting to note that the second interim analysis of CATNON (a phase III trial that randomized adult patients with newly diagnosed non-codeleted anaplastic glioma to either 59.4 Gy radiotherapy (RT) alone; the same RT with concurrent TMZ; the same RT and 12 cycles of adjuvant TMZ; or the same RT with both concurrent TMZ and adjuvant TMZ), found a significant benefit to OS with adjuvant TMZ, but no OS benefit with the addition of concurrent TMZ therapy^1^. Further analysis may result in the amendment of these findings once the data have matured. Whether concurrent TMZ in the setting of newly diagnosed adult glioblastoma treated with RT and adjuvant TMZ is necessary to accrue the survival benefit seen in Stupp in unknown.

Finally, it is difficult to explain the long median OS seen in the TMZ group of CeTeG/NOA-09, which was greater than that of comparable historical groups of patients with tumors with methylated MGMT promoter [CENTRIC trial: 26.4 months, 95% CI 23.9–34.7 (12); Stupp trial 21.7 months, 95% CI not supplied]. The differences might in part be explained by the comparatively high rate of patients with gross total resection and patients with high performance score in CeTeG/NOA-09. Additionally, age was restricted to <70 years in CeTeG/NOA-09, but not in CENTRIC. These features indicate that the results of CeTeG/NOA-09 might not be readily extrapolated and generalizable to an unselected patient population.

One might also be critical of the use of the methylation-specific polymerase chain reaction assay (MSP) for analysis of MGMT promoter methylation, which has since been abandoned (13). It is worth noting that MSP was in wide use when the trial was designed and started recruitment.

In summary, the CeTeG/NOA-09 provides new evidence that dual agent treatment with CCNU may be superior to TMZ alone in the treatment of selected patients with newly diagnosed MGMT promoter methylated GBM. Notably, the decision to study TMZ-CCNU in a treatment paradigm excluding standard-course concurrent TMZ during RT complicates attempts to translate these findings into general neuro-oncology practice. Further study will be required to confirm the integrity and generalizability of these findings.

## Author Contributions

JP, AS, and SD performed literature review and analysis, and were involved in the writing and editing of the manuscript.

### Conflict of Interest

The authors declare that the research was conducted in the absence of any commercial or financial relationships that could be construed as a potential conflict of interest.

## Footnotes

^1^https://meetinglibrary.asco.org/record/173361/abstract
